# Review of the mechanism of cell death resulting from streptozotocin challenge in experimental animals, its practical use and potential risk to humans

**DOI:** 10.1186/2251-6581-12-60

**Published:** 2013-12-23

**Authors:** Chinedum Ogbonnaya Eleazu, Kate Chinedum Eleazu, Sonia Chukwuma, Udeme Nelson Essien

**Affiliations:** 1 Department of Biochemistry, National Root Crops Research Institute, Umudike, Umuahia, Abia State Nigeria; 2 Department of Biochemistry, Michael Okpara University of Agriculture, Umudike, Umuahia, Abia State Nigeria

**Keywords:** Streptozotocin, Animals, Diabetes, Humans, Cell death

## Abstract

Streptozotocin (STZ) (2-deoxy-2-({[methyl(nitroso)amino]carbonyl}amino)-β-D-glucopyranose) is a naturally occurring diabetogenic compound, produced by the soil bacterium *streptomyces achromogenes*, that exhibits broad spectrum of antibacterial properties. Streptozotocin functions as a DNA synthesis inhibitor in both bacterial and mammalian cells. In mammalian cells, the actual mechanism and metabolic targets of STZ toxicity that results in cell death is not known. This review identifies four key areas that explain the mechanism of the cytotoxicity of STZ in mammalian cell lines, investigates the practical aspects of using STZ in experimental animals and the potential risks of its exposure to human health.

**Electronic supplementary material:**

The online version of this article (doi:10.1186/2251-6581-12-60) contains supplementary material, which is available to authorized users.

## Introduction

Streptozotocin (STZ) (2-deoxy-2-(3-methyl-3-nitrosourea)-1-D-glucopyranose) is a naturally occurring compound, produced by the soil bacterium *streptomyces achromogenes*, that exhibits broad spectrum of antibacterial properties [1]. It is a mixture of α- and β-stereoisomers that appear as pale yellow or off-white crystalline powder.

In terms of solubility, it is very soluble in water, ketones and lower alcohols, but slightly soluble in polar organic solvents [2]. Streptozotocin has a molecular formula of C_8_H_15_N_3_O_7_, molecular weight of 265 g/mol and the structure is composed of nitrosourea moiety with a methyl group attached at one end and a glucose molecule at the other end [1].

STZ is a cytotoxic glucose analogue. After its discovery, it was being used as a chemotherapeutic alkylating agent in the treatment of metastasizing pancreatic islet cell tumors and other malignancies [3].

In the year 1963, Rakieten and colleagues reported that STZ is diabetogenic [4]. From that time of discovery till date, STZ has been one of the chemical agents for the induction of diabetes in experimental animals [5].

Streptozotocin functions as a DNA synthesis inhibitor in both bacterial and mammalian cells [6]. In bacterial cells, a specific interaction with cytosine moieties leads to the degradation of the bacterial DNA [7]. Streptozotocin is cytotoxic to pancreatic β-cells and its effects can be seen within seventy two hours after administration depending on the dose administered [8].

In mammalian cells, the mechanism of action of STZ that results in cell death was not fully identified, though it was thought to be a result of DNA and chromosomal damage brought forth by mechanisms involving free radical generation during STZ metabolism [6]. However, after several years of research, the mechanism of action of STZ that results in cell death has been elucidated. The chemical properties of STZ are presented as follows:

A. It is a cytotoxic methyl nitrosourea moiety (N-methyl-N-nitrosourea) attached to the glucose (2-deoxyglucose) molecule.
B. It is a glucosamine derivative
C. It is a toxic beta cell glucose analogue
D. It is a hydrophilic compound
E. It is an alkylating agent
F. STZ is a toxic glucose (Glu) and N-acetyl glucosamine (GlcNAc) analogue that is accumulated preferentially in pancreatic β-cells via GLUT 2 transporter uptake [9].
G. It is relatively stable at pH 7.4 and 37°C at least for up to I hr [10].
H. It has a biological half life of 5–15 minutes [11, 12]
I. When reconstituted into a solution, it can be stored at room temperature or refrigerator but must be used within 12 hrs if stored at room temperature and protected from sunlight.

### Elucidation of the diabetogenic action of STZ

STZ induces diabetes in rats, mice, monkeys, hamsters, rabbits and guinea pigs. The toxic action of STZ involves its uptake into cells. Although nitrosourea compounds are usually lipophilic which makes their uptake by cells very quick, STZ on the contrary is a hydrophilic compound due to hexose substitution which limits its uptake by cells.

The selective pancreatic beta cell toxicity and diabetic condition, resulting from STZ induction, is related to the glucose moiety in its chemical structure which enables STZ to enter the beta cell via the low affinity glucose 2 transporter in the plasma membrane [13] because the β-cells of the pancreas are more active than other cells in taking up glucose and so are more sensitive than other cells to STZ challenge. This statement is validated by the observation that insulin producing cells that do not express this glucose transporter are resistant to STZ toxicity [13] and only become vulnerable to the toxicity of this compound after expression of the GLUT 2 transporter protein in the plasma membrane [13]. Moreover, other cells that express this GLUT 2 transporter such as the hepatocytes and the renal tubular cells are also susceptible to STZ. This explains why experimental animals, inducted with STZ, tend to have renal and liver damage [5, 14]. In addition, non beta cells such as: α-cells as well as the extra-pancreatic parenchyma remain intact after STZ challenge, indicating the beta cell selective properties of STZ [3]. STZ also causes cardiac and adipose tissue damage and increases oxidative stress, inflammation, endothelial dysfunction [15] with the concentrations of the drug or its metabolites in the liver, kidney, intestine and pancreas being consistently higher than those in the plasma.

STZ does not affect the pancreatic beta cells of humans when used in the treatment of islet-cell carcinomas and malignant carcinoid tumors in humans [1]. This resistance of the human beta cells to STZ is attributed to the very low level of the constitutive GLUT 2 transporter expression in the human beta cell [16–20].

### Biochemical basis of the cytotoxicity of STZ that results in cell death (apoptosis/necrosis)

**Figure 1:**
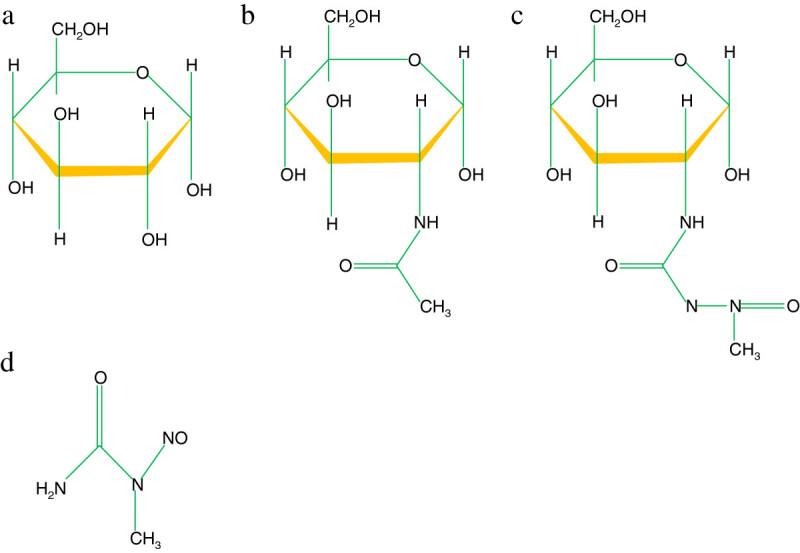
**Structures of (a) glucose (b) N-acetyl glucosamine (c) Streptozotocin and (d) methylnitrosourea.**

STZ is a structural analogue of glucose (Glu) and N-acetyl glucosamine (GlcNAc) (Figure 1). STZ is taken up by pancreatic β-cells via the GLUT 2 transporter where it causes β-cell death by DNA fragmentation due to the nitrosourea moiety as earlier mentioned. Three major pathways associated with cell death are: (i) DNA methylation through the formation of carbonium ion (CH_3_^+^) resulting in the activation of the nuclear enzyme poly ADP-ribose synthetase as part of the cell repair mechanism and consequently, NAD^+^ depletion; (ii) Nitric oxide production (iii) Free radical generation as hydrogen peroxide [9, 21].

#### Methylation of DNA

The DNA methylating activity of the methylnitrosourea moiety of STZ [22], especially at the O^6^ position of guanine, leading to DNA damage with resultant necrosis of the pancreatic beta cells, through the depletion of cellular energy stores, is one explanation for the cell death that results from STZ induction. The resultant activation of polyADP-ribose polymerase (PARP), in an attempt to repair the damaged DNA, depletes the cellular NAD^+^ and consequently, ATP stores as a result of overstimulation of DNA repair mechanisms [23]. Although STZ also methylates proteins, this DNA methylation is most responsible for beta cell death, though STZ methylation of proteins could also contribute to its toxicity to the beta pancreatic cells.

In addition, inhibitors of this poly ADP ribose polymerase such as nicotinamide, inhibit the methylation of DNA by STZ. For example, administration of nicotinamide prior to the induction of STZ in experimental rats, protects the pancreatic cells from the toxic actions of STZ as well as prevents the development of a diabetic state [24]. STZ could also react at other sites of DNA such as the ring nitrogen and exocyclic oxygen atoms of DNA bases, predominantly producing 7-methylguanine, 3-methyladenine which leads to DNA breaks, activates poly-ADP-ribose polymerase and subsequently depletes NAD^+^.

#### Nitric oxide (NO) production

Another possible mechanism of the diabetogenic action of streptozotocin that results in cell death has been attributed to its ability to act as nitric oxide donor in pancreatic cells [25] which inhibits aconitase activity, leading to DNA alkylation and damage [26]. Streptozotocin has been shown to increase the activity of guanyl cyclase and the formation of cGMP, which are characteristic actions of NO. β-cells are particularly sensitive to damage by nitric oxide and free radicals because of their low levels of free radical scavenging enzymes [27].

#### Reactive Oxygen Species (ROS) Production in Oxidative Stress

Oxidative stress is defined as an imbalance between the pro-oxidants and antioxidant defense system of the body as a result of steady state reactive oxygen species. Oxidative stress has recently been shown to be responsible, at least in part, for pancreatic β-cell dysfunction caused by glucose toxicity in hyperglycemia. Several reaction mechanisms are thought to be involved in the genesis of oxidative stress in both diabetic patients and diabetic animals and they include: glucose auto-oxidation, protein glycation, formation of advanced glycation products and the polyol pathway [28, 29]. During these processes, ROS are produced and cause tissue damage [30, 31]. STZ treatment causes significant increase in malonaldehyde but decreases antioxidant enzymes such as: catalase, glutathione peroxidase and superoxide dismutase activities when compared with control animals in experiments. Decreases in antioxidant activities, and simultaneous increases in malonaldehyde (MDA) activities, indicate the susceptibility of pancreas to STZ’s induction of oxidative stress [32, 33].

One important involvement of ROS during STZ metabolism is the production of uric acid as the final product of ATP degradation by xanthine oxidase from hypoxanthine. This reaction generates ROS such as superoxide and hydroxyl radicals emanating from H_2_O_2_ dismutation during hypoxanthine metabolism, accelerating the process of beta cell destruction. This is coupled with the fact that the pancreatic beta cell is devoid of catalase and glutathione peroxidase. The hydrogen peroxide subsequently generates free radicals such as O^2-^ and OH^-^. These reactive compounds can cause peroxidation of lipids, resulting in the formation of hydroperoxy fatty acids and endoperoxides. This increases the formation of malonaldehyde and thromboxane-B2 (TxB2). The accumulation of TxB2 along with thromboxane-A2 (TxA2) can cause platelet aggregation and promote thrombosis [34]. Increased ROS production has also been reported to inhibit aconitase which protects mitochondrial DNA (mtDNA) from degradation [35].

#### Altered NF-κB (nuclear factor kappa-light-chain-enhancer of activated B cells) based cell signaling

A fourth biochemical mechanism for the cytotoxicity of STZ that results in cell death is through altered NF-κB based cell signaling.

STZ selectively inhibits the activity of the glycoside hydrolase *O*-GlcNAcase (enzyme O*-* GlcNAcase catalyzes the cleavage of beta-O-linked GlcNAc (O-GlcNAc) from modified proteins and is a member of the family 84 glycoside hydrolases) in the β-cell, which is responsible for removing *O*-GlcNA from proteins. This causes irreversible *O*-glycosylation of intracellular proteins resulting in β-cell apoptosis [36, 37]. STZ induces beta-cell dysfunction and apoptosis at lower doses while causing beta-cell necrosis at higher doses [38].

Doses (up to 15 mM) of STZ induce pancreatic beta-cell death by inducing apoptosis followed by necrosis at higher doses (up to 30 mM) [39]. Some other researchers reported that STZ challenge (up to 20 mM) caused only apoptotic cell death in other cellular systems [40]. *In vitro* studies using insulin secreting insulinoma cells, keratinocytes and genetically engineered hepatocytes have also shown that STZ (up to 20 mM) causes oxidative stress and apoptosis [40].

However, the actual molecular mechanism and metabolic targets of STZ toxicity in hepatocytes was not known. The mechanism of antineoplastic action of STZ in human hepatoma was also not clearly understood. Using the mitochondrial dehydrogenase based cellular viability MTT (3-(4,5-dimethylthiazol-2-yl)-2,5-diphenyltetrazolium bromide) assay to investigate the dose- and time-dependent effects of STZ on human hepatoma (HepG2 cells) in culture, Haider and Annie [38] showed that STZ induced significant cell death after 48 h of induction with 20 mM of the drug. They also reported that 10 mM STZ resulted in about 40% cell death. At this dose, HepG2 cells exhibit increased ROS and NO production and an increase in lipid peroxidation. The increase in oxidative stress was associated with increased apoptosis of HepG2 cells as evidenced by an increase in caspase-3 activity and reduction in the expression of anti-apoptotic protein, Bcl-2. They further reported that the increased oxidative stress, apoptosis and mitochondrial dysfunction in human hepatoma (HepG2) cells might be associated with altered NF-κB (nuclear factor kappa-light-chain-enhancer of activated B cells, a protein complex that controls the transcription of DNA found in all animal cell types and is involved in cellular response to stimuli, free radicals, oxidized LDL, bacterial or viral antigens) based cell signaling as STZ increases the expression of iNOS (inducible nitric oxide synthase) and translocation of NF-κBp65 (RelA) transcription factor to the nucleus.

## Practical aspects of using Streptozotocin in experimental animals

### Streptozotocin and induction of diabetes in animal species

STZ has proven to be a better diabetogenic agent than alloxan with wider species effectiveness and greater reproducibility. This could be attributed to the fact that STZ is more stable in solution before and after injection in animals than alloxan. In addition, alloxan causes a decrease in hepatic glycogen within 24–72 hours, with greater cytotoxicity due to its conversion to anionic radicals [12] and pancreatic destruction, which insulin partially reverses. Moreover, the STZ model mimics many of the acute and chronic complications of human diabetes and given the established similarities of some of the structural, functional and biochemical abnormalities to human disease, it is an appropriate model to assess the mechanism of diabetes.

When reconstituted into a solution, STZ can be stored at room temperature or refrigerator but must be used within 12 hrs if stored at room temperature and protected from sunlight. Due to streptozotocin’s alleged instability in solution, the typical recommendation is to administer it within 10 minutes after dissolution.

The American Diabetes Association [41] established an etiologic classification of Diabetes mellitus and based on their classification, four groups were proposed: 1) Type 1 (5–10%); 2) Type 2 (90–95%); 3) Other specific types and 4) Gestational. Thus, STZ-induced diabetes belongs to the category of other specific types or Drug (chemical) induced diabetes. However, many researchers conclude that STZ produces type I diabetes mellitus [42].

The type of diabetes induced by STZ is controversial because STZ-hyperglycemia can be similar to either type I or type II diabetes mellitus [42, 43]. The dose of STZ required for inducing diabetes depends on the animal species, age of animal, route of administration, weight of animal, nutritional status [44] and different responses to xenobiotics. For injection in experimental animals and for optimum results, it is best to be administered at fasting state and freshly prepared, dissolved in citrate buffer (pH 4.4-4.5).

Diabetogenic doses vary with species and the optimal doses that have been reported to produce maximum diabetic conditions in various species are: rats (50 to 75 mg/kg ip(intraperitoneal) [14, 25, 34, 45, 46], mice (175 to 200 mg/kg ip or iv (intravenous) [11]; dogs (15 mg/kg for 3 days) [11]. At lower doses, STZ-induced diabetes is not stable, since spontaneous recovery occurs.

In the study carried out by Ventura et al. [9], they reported that a single high dose of 130 or 150 mg/kg bwt or multiple doses of 40 mg/kg bwt produced hyperglycemia similar to type I diabetes and three administrations of multiple low dose generated mild hyperglycemia (250–450 mg/dl), that is similar to type II diabetes in experimental mice.

When administered intravenously, the binding of STZ to its target site is completed within a short time and plasma levels of STZ rapidly decrease within 15 minutes and concentrate in the liver and kidneys [47, 48]. As much as twenty percent of the drug (or metabolites containing an N-nitrosourea group) is metabolized and/or excreted by the kidneys [48]. Thus the biochemical changes observed after 15 minutes of STZ induction are secondary changes and not due to a direct effect of STZ [5]. Complications from any toxic effect of streptozotocin were minimized by carrying out the experiments four to five weeks after the initial streptozotocin injection [45].

Some authors [8] described a triphasic response in blood glucose after streptozotocin administration. According to their study, in the first two hours of STZ challenge, blood glucose rises. This transient hyperglycemia is due to sudden breakdown of liver glycogen. The second phase, starting at about 6 hours after STZ dosing, is a hypoglycemic one, which may be severe enough to lead to death. The third phase, that of permanent hyperglycemia, begins at about 10 to 12 hours after STZ administration. Structural alterations in pancreatic beta cells (total degranulation) occur within 48 h after the administration of streptozocin and last for up to four months [12].

However, in the study carried out by Eleazu et al. [14] and Adeghate and Ponery [49], they reported that the destruction of the insulin secreting β-cells starts three days post STZ administration, reaching its peak at 2 to 4 weeks in rats, leaving less active cells that result in a diabetic state.

In clinical research studies investigating the ameliorating actions of some medicinal plants in diabetic animals induced with STZ, its best to commence administration of the test plants about two weeks post STZ induction or about 11 days after initial hyperglycemic levels since some animals have the ability to return to normoglycemic levels even after initial hyperglycemic levels. Thus if such measures are not taken, one will not know if the transformation to normoglycemic level is as a result of the test plants administered or the animal’s ability to withstand the initial STZ challenge.

### Streptozotocin administration at fasting state

Researchers using diabetic animals for research employ 16–24 hours fasting, but this fasting brings about important changes. These changes tend to affect internal cellular biochemistry and one should therefore expect differences in the effects of preparations on isolated cells, tissue or organs removed from animals that have, or have not been fasted. Fasting has pronounced effects on clinical chemistry analysts and hematology in diabetic animal models. Hypoglycaemia for instance, is more pronounced in fasted animals, therefore STZ should be administered to fed animals to avoid mortalities [50].

Thus researchers using diabetic animal models for their research should consider the effect of fasting for interpretation of their results.

Although, one major reason for subjecting laboratory animals to fasting before blood collection is to reduce variability of some clinical chemistry parameters between feeding and fasting conditions, intestinal physiologic functions and drug-metabolizing enzymes may have some difference under feeding and fasting conditions. Thus, the fasting in animals should be decided on a case by case basis, rather than made uniform for every study.

Administration of 5% glucose solution during the first 24 hours following STZ injection has been reported to prevent early mortalities [51, 52].

### The role of high fat diet (HFD) and low STZ dose in the induction of type 2 diabetes

Injection of STZ (45 and 55 mg kg^-1^ intraperitoneally) after 2 weeks of dietary manipulation has been reported to cause hyperglycemia both in rats fed both normal pellet diet (NPD) and high fat diet [53]. Such rats were reported to be insulin-deficient as compared to the normal rats and exhibited a drastic reduction in the body weight and some of them died within 2 weeks of STZ administration. In addition, insulinotropic (glipizide) and insulin-sensitizing (pioglitazone) agents failed to alter the PGL in these fat-fed/STZ (45 and 55 mg kg^-1^) diabetic rats. Thus, these fat-fed rats with high dose of STZ (45 and 55 mg kg^-1^) resembled more like type I diabetes. The materialization of the disease pattern was achieved by combining the feeding of HFD which produced insulin resistance and low dose of STZ treatment that caused the initial beta cell dysfunction and subsequently the frank hyperglycemia (pre-diabetic state) in non-genetic, out-bred Sprague–Dawley rats. The rats fed with high-fat diet developed obesity, hyperinsulinemia, and insulin resistance, thus limiting the screening of agents on controlling the blood glucose level [53]. Interestingly, the intraperitoneal dose of STZ (35 mg/kg) that produced frank hyperglycemia in HFD-fed rats failed to produce the same in NPD-fed rats. The HFD rat model with low dose of STZ (35 mg kg^-1^) was therefore considered by the authors to represent the pathophysiological state of type 2 diabetes as it was accompanied by marginal increase in body weight in contrast to the catabolic loss of body weight, characteristic of diabetic condition produced by high dose of STZ.

### Streptozotocin and hypernociception

Neuropathy is the most common chronic complication of diabetes mellitus. One of the most elusive symptoms in diabetic neuropathy is pain, characterized by mechanical and thermal hyperalgesia [54]. Hypernociception induced by systemic STZ administration has been widely used as an animal model of diabetic neuropathy.

The pathophysiology of painful diabetes neuropathy is unclear, although it has been associated with impaired peripheral nerve conduction and degeneration of myelinated and unmyelinated fibers [55]. To provide information on underlying mechanisms and to evaluate potential therapies, experimental research on diabetic neuropathy is usually carried out using genetic or chemically induced diabetic animal models. A systemic administration of STZ has been reported to induce hyperalgesia to thermal, mechanical and chemical stimuli [56]. STZ induced hyperalgesia is frequently associated with hyperglycemia because in some studies its development was prevented by insulin treatment [57, 58]. STZ induced hyperalgesia is frequently associated with hyperglycemia because in some studies its development was prevented by insulin treatment [58, 59]. Although these studies suggest that STZ induces painful diabetic neuropathy, it is important to point out that the majority of studies evaluating STZ-induced hyperalgesia only include animals rendered hyperglycemic [60]. In the study carried out by Cunha and colleagues [59], they reported that administration of high dose (40 mg/kg bwt) and low dose (10 or 20 mg/kg bwt) of Streptozotocin produced mechanical hypernociception in all the STZ challenged rats whereas the low dose failed to produce hyperglycemia, suggesting that some other factor other than hyperglycemia could be involved in STZ-induced mechanical hypernociception.

## STZ: human toxicity and care in handling

The interaction with DNA and STZ’s ability to produce cytotoxic effects in animals makes exposure to STZ a significant health and safety threat to laboratory staff, animal handlers, and other personnel who may be subjected to accidental exposure. Due to this health and safety threat, the Institutional Biosafety Committee (IBC) has classified STZ as a reportable hazardous chemical that must be reported on Institutional Animal Care and Use Committee (IACUC) protocols.

Streptozotocin is anticipated to be a human carcinogen. When administered intravenously, STZ has been shown to induce tumors in rat kidney, liver and pancreas as earlier mentioned in addition to neoplastic transformation in primary human kidney cells at doses of 1 mM [61–63]. There are also speculations that STZ can lead to acute complications such as: irritation, nausea, headache, vomiting and chronic complications such as: reproductive disorders, general deterioration of health as well as blindness if in contact with the eyes.

Although there is no scientific evidence to validate such claims, the International Agency for Research on Cancer (IARC) emphasizes that STZ should be regarded for practical purposes as if it were carcinogenic to humans [64] and as such, special care must be taken in preparing STZ for practical use and one of such precautionary measure is to prepare it inside a certified chemical fume hood.

## Conclusion

The cytotoxicity of streptozotocin that results in death can be explained on the basis of DNA methylation through the formation of carbonium ion, resulting in the activation of the nuclear enzyme poly ADP-ribose synthetase and consequently, NAD^+^ depletion; Nitric oxide production, Free radical generation as hydrogen peroxide generation and through altered NF-κB based cell signal transduction pathway. Although there is paucity of information on the carcinogenic effect of STZ on humans, when being used, it should be regarded for practical purposes as if it were carcinogenic to humans. Finally, researchers using diabetic animal models for their research should consider the effect of fasting for the interpretation of their results.

## Authors’ original submitted files for images

Below are the links to the authors’ original submitted files for images. Authors’ original file for figure 1
