# Supplementary figures and images for: Review of the mechanism of cell death resulting from streptozotocin challenge in experimental animals, its practical use and potential risk to humans
Source: J Diabetes Metab Disord. 2013 Dec 23;12:60. doi: 10.1186/2251-6581-12-60 (PMC7962474; doi:10.1186/2251-6581-12-60)

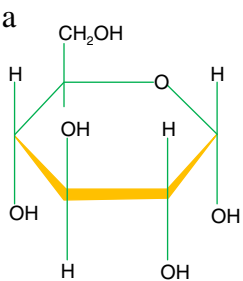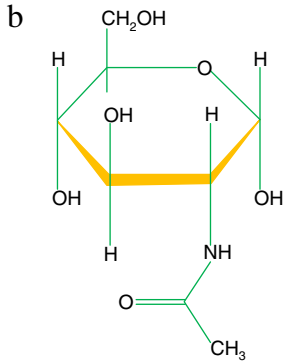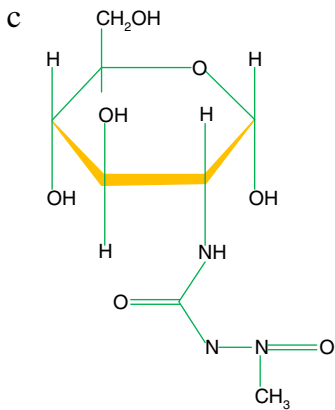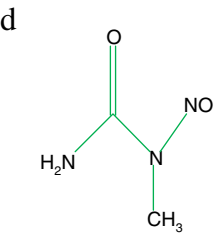

Supplement: Supplementary file 1 — Authors’ original file for figure 1 [file 40200_2013_191_MOESM1_ESM.pdf]
